# P-1057. Candidozyma (Candida) auris in the United States: Insights from the SHEA Research Network

**DOI:** 10.1093/ofid/ofaf695.1252

**Published:** 2026-01-11

**Authors:** Hannah C Lichota, McKenzi King, Rachel L Medernach, Lahari Thotapalli, Mary K Hayden, Sarah E Sansom

**Affiliations:** Rush University Medical Center, Chicago, IL; Rush University Medical Center, Chicago, IL; Rush University Medical Center, Chicago, IL; Rush University Medical Center, Chicago, IL; Rush University Medical Center, Chicago, IL; Rush University Medical Center, Chicago, IL

## Abstract

**Background:**

We queried the Society for Healthcare Epidemiology (SHEA) Research Network (SRN) regarding *C. auris* prevention practices in the United States.

**Table 1:**
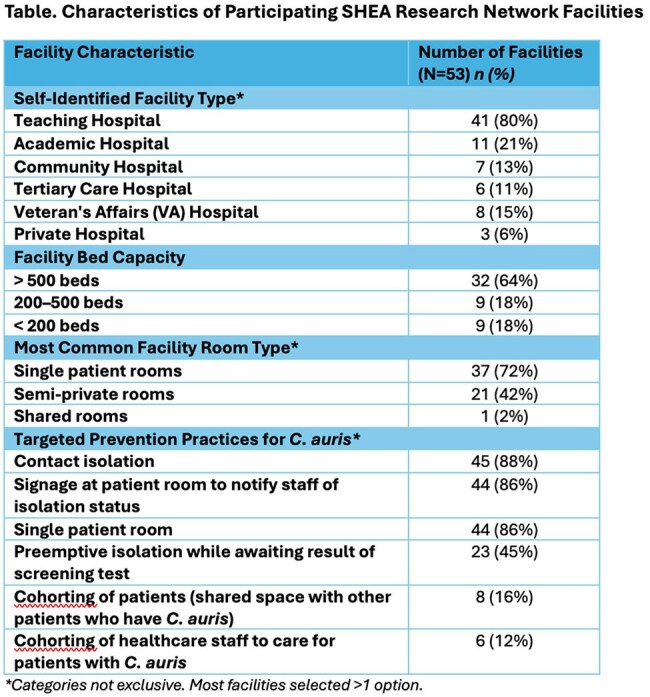
Characteristics of Participating SHEA Research Network Facilities.

**Figure 1:**
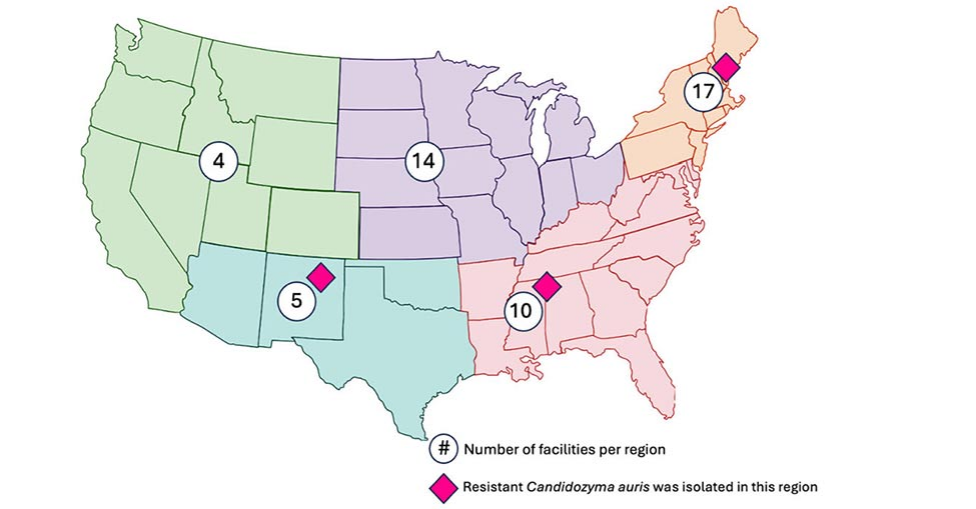
Geographic distribution of SHEA institutions by region. Location and number of participating institutions are shown by geographic region. Pink diamonds indicate a region where survey participants reported a multidrug resistant Candidozyma auris isolate (defined as C. auris isolates with resistance to 3 or more antifungal classes).

**Methods:**

A REDCap survey was distributed by email to SRN institutions in the United States. We assessed institution characteristics, prevention practices, and perceived barriers to *C. auris* prevention.

**Results:**

Responses were received from 53/96 (55%) facilities (Table), with 34/53 (64%) reporting experience with ≥1 *C. auris* case; 32/34 facilities (94%) reported at least one *C. auris* outbreak and 5/34 facilities (14%) had identified a *C. auris* isolate with resistance to ≥3 antifungal classes (Figure). *C. auris* screening was reported in 24/53 (45%) of facilities, including admission screening in 13/24 (54%) facilities and response-based screening in 10/24 (42%). Screening body sites included the axillae (24/24, 100%), groin (23/24, 96%), and anterior nares (7/24, 39%). The most common testing method was polymerase chain reaction-based (17/24, 71%). Prevention practices included patient isolation (45/53, 84%) and enhanced disinfection of shared patient equipment (24/53, 45%) and the healthcare environment (36/53, 68%). The most commonly identified barriers to control of *C. auris* included lack of communication between healthcare facilities (32/53, 60%), lack of microbiologic/diagnostic services (24/53, 45%), and lack of infection control at outside facilities prior to patient transfer (20/53, 37%). The highest priority tools to support *C. auris* prevention were development of effective decolonization regimens (28/53, 53%), standardized protocols for screening (22/53, 42%), and improved communication at time of patient transfer between facilities (20/53, 37%).

**Conclusion:**

Multiple SRN facilities reported firsthand experience with *C. auris*, with a high rate of outbreaks at participating sites. Surveillance was performed in approximately half (45%) of participating facilities, with both admission and response-based screening commonly reported. As *C. auris* becomes increasingly prevalent, additional standardized guidance may help align heterogenous prevention practices.

**Disclosures:**

All Authors: No reported disclosures

